# Independent evolution of the specialized pharyngeal jaw apparatus in cichlid and labrid fishes

**DOI:** 10.1186/1471-2148-7-10

**Published:** 2007-01-30

**Authors:** Kohji Mabuchi, Masaki Miya, Yoichiro Azuma, Mutsumi Nishida

**Affiliations:** 1 Ocean Research Institute, The University of Tokyo, 1-15-1 Minamidai, Nakano-ku, Tokyo 164-8639, Japan; 2 Department of Zoology, Natural History Museum & Institute, Chiba, 955-2 Aoba-cho, Chuo-ku, Chiba 260-8682, Japan

## Abstract

**Background:**

Fishes in the families Cichlidae and Labridae provide good probable examples of vertebrate adaptive radiations. Their spectacular trophic radiations have been widely assumed to be due to structural key innovation in pharyngeal jaw apparatus (PJA), but this idea has never been tested based on a reliable phylogeny. For the first step of evaluating the hypothesis, we investigated the phylogenetic positions of the components of the suborder Labroidei (including Pomacentridae and Embiotocidae in addition to Cichlidae and Labridae) within the Percomorpha, the most diversified (> 15,000 spp) crown clade of teleosts. We examined those based on 78 whole mitochondrial genome sequences (including 12 newly determined sequences) through partitioned Bayesian analyses with concatenated sequences (13,933 bp).

**Results:**

The resultant phylogenies indicated that the Labridae and the remaining three labroid families have diverged basally within the Percomorpha, and monophyly of the suborder was confidently rejected by statistical tests using Bayes factors.

**Conclusion:**

The resultant phylogenies indicated that the specified PJA evolved independently at least twice, once in Labridae and once in the common ancestor of the remaining three labroid families (including the Cichlidae). Because the independent evolution of pharyngeal jaws appears to have been followed by trophic radiations, we consider that our result supports, from the aspect of historical repeatability, the idea that the evolution of the specialized PJA provided these lineages with the morphological potential for their spectacular trophic radiations. The present result will provide a new framework for the study of functional morphology and genetic basis of their PJA.

## Background

**Figure 1:**
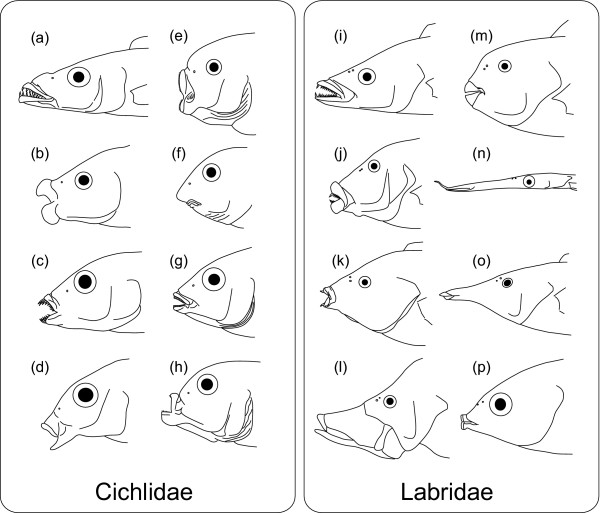
**Diversity of the skull in the Cichlidae (a-h) and Labridae (i-p)**. (a) *Rhamphochromis macrophthalmus*, a piscivore, (b) *Haplochromis euchilus*, a digger in sand, (c) *Labidochromis vellicans*, a picker of small arthropods, (d) *Lethrinops brevis*, a digger in sand, (e) *Petrotilapia tridentiger*, a rock scraper, (f) *Labeotropheus fuelleborni*, an algal-eating rock scraper, (g) *Haplochromis similis*, a leaf chopper, (h) *Genyochromis mento*, a scale eater, (i) *Cheilinus celebicus*, feeds on small fishes and invertebrates, (j) *Hemigymnus melapterus*, feeds on invertebrates in sand, (k) *Anampses geographicus*, feeds on small hard-shelled invertebrates, (l) *Epibulus insidiator*, engulfs crustaceans and small fishes, (m) *Chlorurus microrhinos*, feeds on the epilithic algal matrix of coral reefs, (n) *Siphonognathus argyrophanes*, feeds on small invertebrates picked from weeds or the substratum, (o) *Gomphosus varius*, feeds on small benthic crustaceans, (p) *Labrichthys unilineatus*, a coral-polyp eater. Drawings of cichlids modified from Fryer & Iles [59].

Fishes of the families Cichlidae and Labridae [1] (including scarids and odacids as subgroups) represent good probable examples of vertebrate adaptive radiations [2,3]. Although the two families inhabit different aquatic environments (freshwater cichlids vs marine labrids), both families have radiated into several hundreds to over a thousand of species exhibiting various feeding modes (Fig. 1). In terms of species richness, cichlids exceed labrids (1478 spp. vs 576 spp.; calculated from the data from FishBase [4], whereas labrid morphological diversity is comparable to that of the cichlids in terms of feeding modes, as represented by the wide variety of their skull forms (Fig. 1) [5]. Indeed, biomechanical studies of lower jaw shapes [6,7] quantitatively demonstrated that the labrid functional diversity in lower jaw was well comparable to that of all ray-finned fishes.

**Figure 2:**
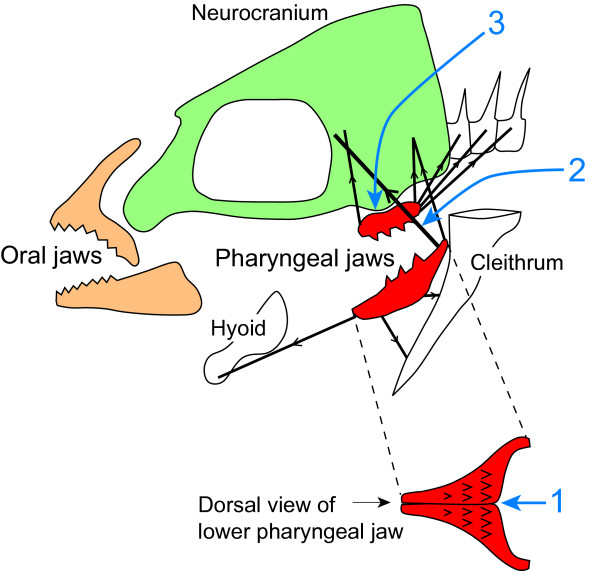
**Diagrammatic representation of the principal components of the specifically modified PJA of cichlids**. Red elements are the upper and lower pharyngeal jaws. The muscles organizing the PJA (pharyngeal jaw apparatus) are represented as black thick lines, and the principal directions of force has been indicated by arrows. The drawing modified from Liem & Greenwood [32]. Numbers indicate three major features of the specialized "labroid" PJA: 1) the left and right lower jaw elements are fused into a single structure, 2) the lower jaw is suspended in a muscular sling that runs from the neurocranium to the posterior muscular arms of the lower jaw, and 3) the upper jaw elements have a diarthrotic articulation with the underside of the neurocranium.

The dramatic radiation of the cichlid and labrid fishes is widely believed to result from the "key innovation" of a unique pharyngeal jaw apparatus (PJA). Generalized percomorph fishes, from which the cichlids and labrids were derived, have upper and lower "pharyngeal jaws" within the throat, which function chiefly to transport food into the esophagus, whereas the "oral jaws" have a task of both collecting and manipulating (crushing and processing) food. In the cichlid and labrid fishes, the pharyngeal jaws and attached muscles (constituting PJA) was modified into a derived condition with three major features: 1) the left and right lower jaw elements are fused into a single structure, 2) the lower jaw is suspended in a muscular sling that runs from the neurocranium to the posterior muscular arms of the lower jaw, and 3) the upper jaw elements have a diarthrotic articulation with the underside of the neurocranium (Fig. 2) [8]. The unique condition of the PJA is assumed to allow efficient manipulation (particularly strong bite) of food [8-10]. Liem [9] has suggested that the evolution of the uniquely modified PJA in cichlids and labrids freed the oral jaws from the task of food manipulation, allowing it to diversify into various food-collecting modes (see Fig. 1).

Although this scenario is one of the most well-known examples of an evolutionary key innovation (cited in Futuyma's [11] "*Evolutionary Biology*", the most widely-used textbook in the field), it has never been evaluated adequately. Unfortunately, the key innovation hypothesis on the specialized PJA has been proposed being coupled with a taxonomic hypothesis that has widely been accepted ever since: the family Labridae and Cichlidae have been classified into the suborder Labroidei, together with Pomacentridae and Embiotocidae, based on the sharing of the specialized PJA [10,12]. According to Stiassny & Jensen [12], the specialized PJA has seven morphological features including the above three major ones. None of them is, however, unique to labroid fishes, although there is no other group known to have them all [13]. Moreover, there is no independent, corroborative evidence for a monophyletic Labroidei outside of the specialized PJA [12,13]. This situation has led some evolutionary biologists to doubt not only the taxonomic hypothesis but also the key innovation hypothesis (*e.g. *[14]). The doubt about the latter hypothesis is a little rash, however, because appropriateness of the evolutionary hypothesis essentially does not depend on that of the appended taxonomic hypothesis.

A discussion of the specialized PJA as a key innovation, however, hinges, first of all, upon a phylogenetic hypothesis independent of pharyngeal characters as below. The widely-accepted taxonomic hypothesis (monophyletic Labroidei) assumed that the evolution of the specialized PJA occurred only once during the percomorph evolution, a situation that has prevented the famous evolutionary example from being examined from the aspect of historical repeatability. The traditional taxonomic hypothesis based on the sharing of the specialized PJA, however, needs reconsideration. Phylogeny independent of the PJA may come to demonstrate multiple origins of the specialized PJA followed by trophic radiations, which support the key innovation hypothesis at least from the aspect of the historical repeatability. The hypothesis, however, must be tested statistically by comparing the diversity of the clades with the trait to that of the sister taxa without that trait [15,16] when the true sister taxa are found in a future study.

For the first step of evaluating the key innovation hypothesis on the specialized PJA, we examined labroid monophyly based on 78 whole mitochondrial genome sequences from a variety of the Perciformes and related other ordinal taxa (collectively called "Percomorpha"). Usefulness of whole mitochondrial genome sequences (ca. 16,000 bp) for resolving higher-level phylogenetic relationships of teleost has been demonstrated in several studies [*e.g. *[17-19]]. Although non-monophyly of the labroid families have been suggested in previous studies [14,20,21], limited sequence data used in these studies (2,222 bp at longest) have precluded them from drawing definite conclusions, and no statistical test has been conducted for corroborating that hypothesis. Our analysis used the whole mitochondrial genome sequences (>13,000 bp per species), and confirmed the independent origins of the two diversified families by statistical tests using Bayes factors.

## Results

**Figure 3:**
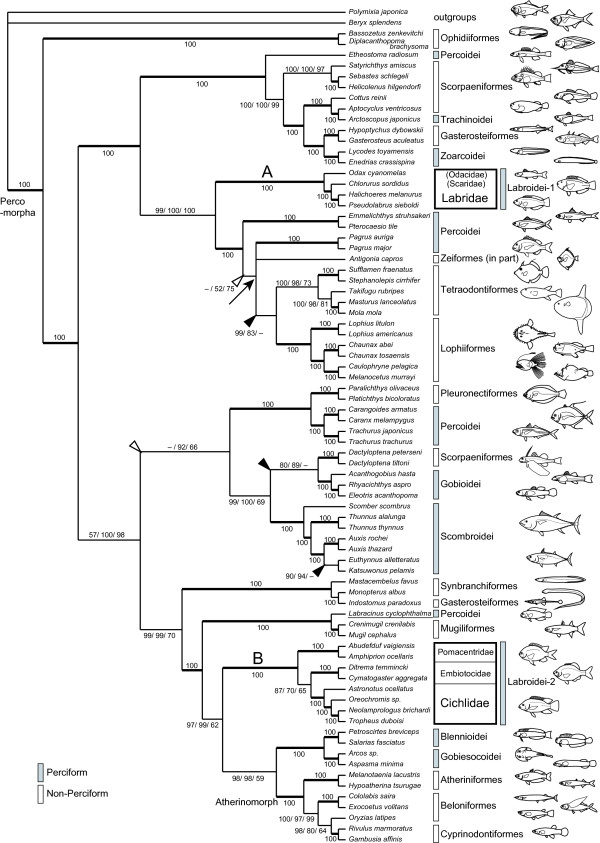
**Phylogenetic relationships among Labroid families, based on whole mitochondrial DNA sequences**. Shown is the 50% majority rule consensus tree of the 4,700 pooled trees from two independent Bayesian analyses for dataset #2 (3rd codons RY-coded). The dataset comprises unambiguously aligned nucleotide sequences of 13,393 bp from 76 percomorphs and two outgroups; we set five partitions (1st, 2nd and 3rd codon positions from 12 protein-coding genes plus tRNA and rRNA genes). Partitioned Bayesian analyses were conducted using MRBAYES 3.1.2 [53], with the best-fit model of sequence evolution [ref. 54; GTR + I + Γ] being set each partition and all model parameters variable and unlinked across partitions. Numerals beside internal branches indicate Bayesian posterior probabilities (PPs) (shown as percentages) for dataset #1/#2/#3. Single numerals are given when analyses for all the datasets have shown the same values, and clades denoted by broad lines indicate those supported by 100% PPs in the all datasets. An unresolved trichotomy is indicated by an arrow, and topological incongruities among the datasets are denoted by open arrowheads (dataset #1 vs. #2) and filled arrowheads (dataset #1 vs. #3). Note that the species of the Labridae and those of the remaining three labroid families (Cichlidae, Pomacentridae, and Embiotocidae) form different monophyletic groups, respectively.

The final DNA alignment contains 13,933 nucleotide sites for the 78 taxa listed in Table 1. Of the sites, 9,037 were variable and 7,832 informative under the parsimony criterion. Partitioned Bayesian analyses based on three differently-weighted datasets, #1, #2 (3rd codons RY-coded), and #3 (3rd codons excluded), recovered nearly identical topologies. Figure 3 shows a 50% majority rule consensus tree of the 4,700 pooled trees from two independent Bayesian analyses for dataset #2. It was fully bifurcated with the exception of an unresolved trichotomy denoted by an arrow, and most internal branches were supported by 100% posterior probabilities (PPs). Topological differences among results from the three datasets were shown only in the trichotomy and five internal branches denoted by arrowheads in Fig. 3.

**Table 1:** List of species used in this study, with DDBJ/EMBL/GenBank Accession Numbers. Classifications follow Nelson [23].

Order	Family	Species	Accession No.
Outgroups			
Polymixiiformes	Polymixiidae	*Polymixia japonica*	[DDBJ: AB034826]
Beryciformes	Berycidae	*Beryx splendens*	[DDBJ: AP002939]
Percomorpha *sensu *Miya *et al*. [18]			
Ophidiiformes	Ophidiidae	*Bassozetus zenkevitchi*	[DDBJ: AP004405]
	Bythitidae	*Diplacanthopoma brachysoma*	[DDBJ: AP004408]
Lophiiformes	Lophiidae	*Lophius americanus*	[DDBJ: AP004414]
		*Lophius litulon*	[DDBJ: AP004413]
	Chaunacidae	*Chaunax abei*	[DDBJ: AP004415]
		*Chaunax tosaensis*	[DDBJ: AP004416]
	Caulophrynidae	*Caulophryne pelagica*	[DDBJ: AP004417]
	Melanocetidae	*Melanocetus murrayi*	[DDBJ: AP004418]
Mugiliformes	Mugilidae	*Mugil cephalus*	[DDBJ: AP002930]
		*Crenimugil crenilabis*	[DDBJ: AP002931]
Atheriniformes	Melanotaeniidae	*Melanotaenia lacustris*	[DDBJ: AP004419]
	Atherinidae	*Hypoatherina tsurugae*	[DDBJ: AP004420]
Beloniformes	Adrianichthyidae	*Oryzias latipes*	[DDBJ: AP004421]
	Scomberesocidae	*Cololabis saira*	[DDBJ: AP002932]
	Exocoetidae	*Exocoetus volitans*	[DDBJ: AP002933]
Cyprinodontiformes	Aplocheilidae	*Kryptolebias marmoratus*	[GenBank: AF283503]
	Poeciliidae	*Gambusia affinis*	[DDBJ: AP004422]
Zeiformes	Caproidae	*Antigonia capros*	[DDBJ: AP002943]
Gasterosteiformes	Hypoptychidae	*Hypoptychus dybowskii*	[DDBJ: AP004437]
	Gasterosteidae	*Gasterosteus aculeatus*	[DDBJ: AP002944]
	Indostomidae	*Indostomus paradoxus*	[DDBJ: AP004438]
Synbranchiformes	Synbranchidae	*Monopterus albus*	[DDBJ: AP002945]
	Mastacembelidae	*Mastacembelus favus*	[DDBJ: AP002946]
Scorpaeniformes	Dactylopteridae	*Dactyloptena tiltoni*	[DDBJ: AP004440]
		*Dactyloptena peterseni*	[DDBJ: AP002947]
	Scorpaenidae	*Helicolenus hilgendorfi*	[DDBJ: AP002948]
		*Sebastes schlegeli*	[GenBank: AY491978]
	Triglidae	*Satyrichthys amiscus*	[DDBJ: AP004441]
	Cottidae	*Cottus reinii*	[DDBJ: AP004442]
	Cyclopteridae	*Aptocyclus ventricosus*	[DDBJ: AP004443]
Perciformes	Pseudochromidae	*Labracinus cyclophthalma*	[DDBJ: AP009125]
	Percidae	*Etheostoma radiosum*	[GenBank: AY341348]
	Carangidae	*Carangoides armatus*	[DDBJ: AP004444]
		*Caranx melampygus*	[DDBJ: AP004445]
		*Trachurus japonicus*	[DDBJ: AP003091]
		*Trachurus trachurus*	[DDBJ: AB108498]
	Emmelichthyidae	*Emmelichthys struhsakeri*	[DDBJ: AP004446]
	Lutjanidae	*Pterocaesio tile*	[DDBJ: AP004447]
	Sparidae	*Pagrus auriga*	[DDBJ: AB124801]
		*Pagrus major*	[DDBJ: AP002949]
	Cichlidae	*Oreochromis *sp.	[DDBJ: AP009126]
		*Neolamprologus brichardi*	[DDBJ: AP006014]
		*Tropheus duboisi*	[DDBJ: AP006015]
		*Astronotus ocellatus*	[DDBJ: AP009127]
	Embiotocidae	*Cymatogaster aggregata*	[DDBJ: AP009128]
		*Ditrema temmincki*	[DDBJ: AP009129]
	Pomacentridae	*Abudefduf vaigiensis*	[DDBJ: AP006016]
		*Amphiprion ocellaris*	[DDBJ: AP006017]
	Labridae	*Pseudolabrus sieboldi*	[DDBJ: AP006019]
		*Halichoeres melanurus*	[DDBJ: AP006018]
	Odacidae	*Odax cyanomelas*	[DDBJ: AP009130]
	Scaridae	*Chlorurus sordidus*	[DDBJ: AP006567]
	Zoarcidae	*Lycodes toyamensis*	[DDBJ: AP004448]
	Pholidae	*Enedrias crassispina*	[DDBJ: AP004449]
	Trichodontidae	*Arctoscopus japonicus*	[DDBJ: AP003090]
	Blennidae	*Petroscirtes breviceps*	[DDBJ: AP004450]
		*Salarias fasciatus*	[DDBJ: AP004451]
	Gobiesocidae	*Arcos *sp.	[DDBJ: AP004452]
		*Aspasma minima*	[DDBJ: AP004453]
	Rhyacichthyidae	*Rhyacichthys aspro*	[DDBJ: AP004454]
	Eleotridae	*Eleotris acanthopoma*	[DDBJ: AP004455]
	Gobiidae	*Acanthogobius hasta*	[GenBank: AY486321]
	Scombridae	*Auxis rochei*	[DDBJ: AB103467]
		*Auxis thazard*	[DDBJ: AB105447]
		*Euthynnus alletteratus*	[DDBJ: AB099716]
		*Katsuwonus pelamis*	[DDBJ: AB101290]
		*Scomber scombrus*	[DDBJ: AB120717]
		*Thunnus alalunga*	[DDBJ: AB101291]
		*Thunnus thynnus thynnus*	[GenBank: AY302574]
Pleuronectiformes	Paralicthyidae	*Paralichthys olivaceus*	[DDBJ: AB028664]
	Pleuronectidae	*Platichthys bicoloratus*	[DDBJ: AP002951]
Tetraodontiformes	Balistidae	*Sufflamen fraenatus*	[DDBJ: AP004456]
	Monacanthidae	*Stephanolepis cirrhifer*	[DDBJ: AP002952]
	Tetraodontidae	*Takifugu rubripes*	[EMBL: AJ421455]
	Molidae	*Masturus lanceolatus*	[DDBJ: AP006239]
		*Mola mola*	[DDBJ: AP006238]

In the resultant trees, every family of the suborder Labroidei (Cichlidae, Pomacentridae, Embiotocidae, and Labridae) was monophyletic with 100% PPs, and moreover, the family Labridae (clade A) was phylogenetically distant from the remaining three labroid families forming clade B. Monophyly of the Labroidei comprised of all the four families was confidently rejected by a statistical test using Bayes factor (2 ln = 540.28). The family Pseudochromidae (represented by *Labracinus cyclophthalma*, underlined in Fig. 3), which was sometimes thought to be a possible sister to the Labridae based on larval morphology [22], was placed not close to the Labridae (clade A), but more closely related to the clade B.

## Discussion

### Percomorpha phylogeny

Overall relationships among the percomorph fishes examined here were quite similar to those obtained by unweighted and weighted maximum-parsimony analyses in Miya *et al*. [18], except for the newly added fishes, such as labroids. Although the phylogenies recovered many interesting inter-subordinal relationships that were not congruent with the traditional classification, sparse taxon sampling outside the Labroidei precludes further discussions.

### Labroid phylogeny

The resultant trees and the statistical test with Bayes factor demonstrated that the Labridae and the remaining three labroid families including Cichlidae have independent origins within the Percomorpha. Based on molecular analyses, various relationships among the labroid families have been so far proposed [14,20,21]. Our analysis demonstrated entirely different relationships from the various ones with high statistical support, whereas none of the previous relationships was supported with high statistical value, probably owing to short sequences (2,222 bp at longest). The present analyses were conducted based on the long nucleotide sequences more than six times the lengths of those so far used (13,933 bp).

In spite of the high statistical supports, we must keep in mind that the sister relationships obtained here are highly tentative because of the sparse taxon sampling (76 species from 53 families) compared to the tremendous taxonomic diversity of the Percomorpha (*ca*. 14,000 species in 251 families; calculated from Nelson [23]). That is to say, although the present phylogeny demonstrated that the three labroid families, Cichlidae, Pomacentridae, and Embiotocidae, formed a well-supported monophyletic group (clade B) without non-labroid taxa, there remains a possibility that some non-labroid taxa would break into the group when more extensive taxon sampling is conducted. In the same way, there remains not a few possibilities that some yet-to-be-analyzed taxa (*ca. *200 families left) would become sister group of the "labroid" taxa. To evaluate these possibilities, we need to conduct analyses with more extensive taxon sampling. In doing so, we would be able to evaluate "key innovation" hypothesis in a more rigorous manner using statistical test [15,16].

### Implications of the revised labroid phylogeny

The present result supports, from the aspect of historical repeatability, the famous (but never evaluated) evolutionary hypothesis that the dramatic labroid radiations in trophic ecology are due to evolution of specialized pharyngeal jaw apparatus (PJA). Based on the present phylogeny, a single origin of the specialized PJA in a common ancestor of the suborder Labroidei is unlikely, because such an evolutionary scenario requires subsequent losses of the complex structures in a wide variety of percomorphs. Rather, it is plausible that the specialized PJA has evolved twice independently in the Percomorpha history, in a common ancestor of the Labridae (clade A) and that of the Cichlidae, Pomacentridae, and Embiotocidae (clade B). Interestingly, evolutionary radiations in jaw forms and head shape (see Fig. 1) have occurred within both of the lineages with specialized PJA: one in the Labridae, and the other in the Cichlidae. The independent occurrence of such evolutionary succession (evolution of specialized PJA followed by trophic radiation) appears to support the idea that the evolution of the specialized PJA provided these lineages with the morphological "potential" for trophic radiations.

Despite their specialized PJA, the Pomacentridae and Embiotocidae seem not to have experienced clear trophic radiation. This may reflect the context-dependence of a key trait's effect on diversification [24]. There are many potential factors that could influence how a key trait affects diversification. De Queiroz [24] categorized these factors roughly into three types: (1) other taxa, (2) other traits of the group itself, and (3) the physical environment. Evolutionary radiations in African Great Lake cichlids are supposedly facilitated by colonization of novel habitats (newly created lakes) without other competitive taxa [25]. The Pomacentridae and Embiotocidae, both of which inhabit coastal marine waters, may not have had such opportunity to colonize novel habitats without competitors.

### Evolution of the specialized "labroid" PJA

It is interesting fact that the complex "labroid" PJA evolved multiply in the Percomorpha history (other than the two lineages recognized here, two fossil labroid families have been recognized so far from the lower Middle Eocene [*ca*. 50 million years ago] of Monte Bolca, Italy, together with the oldest fossil records of the Labridae and Pomacentridae [26-29]). Is the acquisition of the "successful" PJA not so difficult for percomorph fishes, like parallel evolution of trophic morphologies in African Great Lake cichlids [30]? According to Stiassny & Jensen [12], typical labroid PJA has seven features (three of them shown in Fig. 2). Interestingly, none of them is unique to labroid fishes ([12]; *e.g. *fused lower pharyngeal jaw is also shown in several families of Perciformes and members of Beloniformes), though there is no other group known to have them all [13]. In addition to it, it seems that such features can be accomplished by a very simple change in ontogenetic mechanism (*e.g. *fusion of lower pharyngeal jaws into one unit) [9]. The labroid PJA is therefore considered to have been achieved through the unique combination of several commonplace and slight morphological modifications. It will be worthwhile to study the molecular mechanisms underlying the morphological modifications, which would reveal molecular basis for the probable key innovations. According to Hulsey *et al. *[31], more than ten genes have been so far recognized which putatively influence teleost pharyngeal jaw.

The present result will provide a new view point for functional morphology of the unique organ (the "labroid" PJA). Although many works on the trophic apparatus have been so far published [*e.g. *[8-10,12,32-35]], comparison between labrid and other labroid fishes (including cichlids) as evolutionary independent lineages has never been conducted. Careful comparisons, particularly based on more extensive phylogenies including labroid and non-labroid fishes possessing "partly" specialized PJA (*e.g. *members of Beloniformes), will shed light on overlooked evolutionary implications.

## Conclusion

The phylogenetic analyses of whole mitochondrial DNAs from various labroid and non-labroid "percomorph" fishes revealed that the Labridae and the remaining three labroid families have diverged basally within Percomorpha, indicating that the specified "labroid" PJA evolved independently at least twice, once in Labridae and once in the common ancestor of the remaining three labroid families (including the Cichlidae). Because both of the evolution appear to be followed by trophic radiations, we consider that our result supports the idea that the evolution of the specialized PJA provided these lineages with the morphological potential for their spectacular trophic radiations.

The present result will provide a new view point for functional morphology of the unique organ: although many works on the specialized PJA have been so far published, comparison between labrid and other labroid fishes (including cichlids) as evolutionary independent lineages has never been conducted. Careful comparisons, particularly based on more extensive phylogenies, will shed light on overlooked evolutionary implications.

## Methods

### Taxon sampling

To examine monophyly of suborder Labroidei, we used all four labroid families (Labridae, Cichlidae, Pomacentridae, and Embiotocidae) and a possible sister family for Labridae (Pseudochromidae) in our analysis. In addition, we used all available whole mitochondrial genome (mitogenome) sequences from the Percomorpha (sensu Miya *et al. *[18]) in the database (NCBI Organelle Genome Resources) [36] in the analyses. We chose two basal acanthomorph (but not percomorph) taxa, *Polymixia japonica *and *Beryx splendens*, as collective outgroups to root the trees. A list of taxa examined in this study is provided in Table 1 along with DDBJ/EMBL/GenBank accession.

### DNA extraction, PCR, and sequencing

We amplified whole mitogenome sequences for the 11 labroids plus a single non-labroid species (*Labracinus cyclophthalma*: Pseudochromidae) using a long PCR technique [37]. We used six fish-versatile long PCR primers in the following four combinations (S-LA-16S-L + S-LA-16S-H; L2508-16S + H1065-12S; L2508-16S + H12293-Leu; L12321-Leu + S-LA-16S-H; for locations and sequences of these primers, see Miya & Nishida [17,38], Inoue *et al*. [39,40], Ishiguro *et al*. [41], Kawaguchi *et al*. [42]) so as to amplify the entire mitogenome in two reactions. Long PCR reaction conditions followed Miya & Nishida [38]. Dilution of the long PCR products was with sterile water (1:10–100) served for subsequent uses as short PCR templates.

We used a total of 162 fish-versatile PCR primers in various combinations to amplify contiguous, overlapping segments of the entire mitogenome for each of the 12 species (for locations and sequences of the primers, see Miya & Nishida [17,38], Inoue *et al*. [39,40,43-45]; Ishiguro *et al*. [41], Kawaguchi *et al*. [42]). Short PCR reaction conditions followed Miya & Nishida [38].

Double-stranded short PCR products, purified using a Pre-Sequencing kit (USB), subsequently served for direct cycle sequencing with dye-labeled terminators (Applied Biosystems and Amersham Pharmacia) with the same primers for the short PCRs. All sequencing reactions were performed according to the manufacture's instructions. Labeled fragments were analyzed on model 373/377/3100 sequencers (Applied Biosystems).

### Sequences editing and alignment

The sequence electropherograms were edited with the computer programs EDITVIEW (Version 1.01; Applied Biosystems). AUTOASSEMBLER (Version 2.1; Applied Biosystems) and DNASIS (Version 3.2; Hitachi Software Engineering) were used to concatenate the consensus mitogenomic sequences. Then, sequences were exported to phylogenetic software programs. To build our character matrix, we combined the 12 newly determined sequences with the 66 previously published sequences.

For each individual protein-coding gene, we manually aligned the sequences for the 78 species, with respect to the translated amino acid sequence, using MACCLADE [46]. All stop codons and gaps were excluded from the subsequent phylogenetic analyses, as well as ambiguously aligned regions. The ND6 gene was also excluded because of its heterogeneous base composition and consistently poor phylogenetic performance [17]. The 22 tRNA genes were also aligned manually, and ambiguous and gap alignment were excluded from the phylogenetic analyses. The 12S and 16S rRNA sequences were aligned using the software PROALIGN (Version 0.5) [47], and default setting parameters. Regions with posterior probabilities of ≤ 50% were excluded from the subsequent phylogenetic analyses.

### Phylogenetic analysis

When using a mitogenomic dataset for a particular taxonomic sampling, Simmons & Miya [48] empirically demonstrated that Bayesian analysis [49-52] is the most efficient character-based method for accurately reconstructing phylogeny. Following their recommendations, we used this method for constructing the labroid phylogeny.

Partitioned Bayesian phylogenetic analyses were performed with MRBAYES (Version 3.04 b) [53] for three different character matrices. The first matrix (dataset #1, 13,933 positions) includes concatenated nucleotide sequences from 12 protein-coding genes (10,758 positions), 22 transfer RNA genes (1,406 positions) and the two ribosomal RNA genes (1,769 positions). The second (dataset #2, 13,933 positions) includes the same set of characters with 3rd codon positions of the protein-coding genes converted into purine (R) and pyrimidine (Y), and the third matrix (dataset #3, 10,347 positions) with the 3rd codon positions excluded. Assuming that functional constraints on sequence evolution are more similar within codon positions (or types of molecules) across genes than across codon positions (or types of molecules) within genes, five partitions (1st, 2nd and 3rd codon positions of protein-coding genes, tRNA genes and rRNA genes) were set for the datasets #1 and #2, and four partitions (no third codon positions included) were done for dataset #3.

The general time reversible model with some sites assumed to be invariable and with variable sites assumed to follow a discrete gamma distribution [ref. [54]; GTR + I + Γ] was used for model of sequence evolution, as it was selected as best-fitting model with MRMODELTEST (Version 2) [55] for each partition except for positions with the RY-coding. We assumed that all of the model parameters were unlinked and rate multipliers were variable across partitions. For 3rd codon positions in the dataset #2, we used arbitrarily "A" and "C" instead of "R" and "Y" and set a single rate category (lset nst = 1) instead of six (lset nst = 6) to allow the program to estimate only transversional changes between purine (R) and pyrimidine (Y) nucleotides. MrBayes unnecessarily estimated transitional changes (A ↔ G or C ↔ T) when RY-coding was employed, which simply imposed fixed nucleotide compositions on R (A + G) and Y (C + T) during the calculation.

For each of the three matrices, two independent Bayesian analyses with Markov chain Monte Carlo (MCMC) process of 3,000,000 generations were performed. Parameter values and trees were sampled every 1,000 generations, and the samples before the convergence of the Markov chain were discarded for each run. The remaining samples from the two independent run were combined into a single file with a total of 5,200, 4,700 and 5,520 phylogenetic trees, respectively. The combined tree files were then imported into PAUP (Version 4.0b10) [56] to compute the 50% majority rule consensus trees. The percentages for the branches in the consensus trees represent the Bayesian posterior probabilities, which are the rough equivalent of a maximum likelihood search with bootstrapping [53].

### Testing an alternative phylogenetic hypothesis

We tested an alternative hypothesis for labroid phylogeny using Bayes factors. Constrained Bayesian trees (imposing labroid monophyly on the trees) were estimated with MRBAYES (Version 3.1.2) [53] and harmonic means of log likelihood scores (calculated using "sump" command) were compared between constrained and unconstrained trees. Two independent partitioned Bayesian analyses with MCMC process of 3,000,000 generations were performed based on the dataset #2 (RY coding), which effectively removes likely "noise" from the dataset and avoids the apparent lack of signal by retaining all available positions in the dataset. Parameters and trees were sampled in the same way as described above, being combined into a single file with a total of 5,200 trees. The alternative trees were then compared using a Bayesian approach with Bayes factors [57], the traditional criterion of 2 ln Bayes factor of ≥ 10 being used as very strong evidence against the alternative hypothesis [58].

## Authors' contributions

KM, MM and MN designed the study, and all authors were involved in sampling. KM, MM and YA carried out the molecular work, and KM analyzed the data and drafted the manuscript. MM and MN helped draft the manuscript. All authors read and approved the final manuscript.
